# Paraneoplastic Opsoclonus Myoclonus in a Patient with Pancreatic Adenocarcinoma

**DOI:** 10.1155/2019/3601026

**Published:** 2019-04-16

**Authors:** Divine C. Nwafor, Ashley B. Petrone, Joseph M. Collins, Amelia K. Adcock

**Affiliations:** ^1^Department of Neuroscience, School of Medicine, West Virginia University, Morgantown, WV, USA; ^2^Department of Pathology, Anatomy and Laboratory Medicine, School of Medicine, West Virginia University, School of Medicine, Morgantown, WV, USA; ^3^Department of Radiology, Mayo Clinic Arizona, Scottsdale, AZ, USA; ^4^Department of Neurology, School of Medicine, West Virginia University, Morgantown, WV, USA

## Abstract

Opsoclonus myoclonus syndrome (OMS) is an extremely rare neurological syndrome typically affecting as few as 1 in 10,000,000 people annually. OMS is characterized by the presence of involuntary, saccadic eye movements, as well as ataxia, including gait incoordination, rigidity, and tremor. The origin of OMS is unclear, but a significant percentage of OMS cases are indicative of an underlying malignancy, most commonly neuroblastoma and small cell lung cancer. Here we describe an adult patient with OMS, who was ultimately diagnosed with a small ductal adenocarcinoma of the pancreas. To our knowledge, this is the third published report of an association between OMS and pancreatic malignancy, and the only case where the pancreatic malignancy was detected prior to metastasis or autopsy at death. This case report highlights the importance of careful, aggressive malignancy screening with OMS, as the pancreatic duct cut-off sign may be overlooked if pancreatic malignancy is not suspected.

## 1. Introduction

Opsoclonus myoclonus syndrome (OMS) is an extremely rare autoimmune neurological syndrome typically affecting as few as 1 in 10,000,000 people annually. The etiology of OMS is thought to be paraneoplastic (e.g., malignancies) or nonparaneoplastic (e.g., idiopathic, parainfectious) in origin [1–3]. The clinical presentation of OMS is characterized by the presence of involuntary and saccadic eye movements, as well as ataxia, including gait incoordination, rigidity, and tremor. The pathogenesis of OMS remains unclear; however, it has been proposed that it could be the result of dysfunctional motor neurons, specifically pontine omnipause neurons and/or cerebellar Purkinje cells [4]. Twenty to forty percent of OMS cases in adults and children are indicative of an underlying malignancy [2, 5, 6]. The tumors most frequently associated with OMS in children and adults are neuroblastoma and small cell lung cancer (SCLC), respectively [7]. We describe an adult patient with OMS, who was ultimately diagnosed with ductal adenocarcinoma of the pancreas. To our knowledge, this is the third published report of an association between OMS and pancreatic malignancy, and the only case where the pancreatic malignancy was detected prior to metastasis or autopsy at death [8, 9].

## 2. Case Presentation

Here we describe, a 72-year-old female with a history of hypothyroidism, hyperlipidemia, hypertension, and 50 pack years of smoking, who presented to an outside facility with a 30-pound weight loss, severe nausea, emesis, mild imbalance which graduated to bedbound instability, and involuntary body “shakes” progressing over 6 months. Initial investigations at an outside facility, including contrast enhanced-MRI imaging of the entire neuroaxis, EEG, colonoscopy, and basic hematologic and chemistry panels, were all normal. The only initial abnormal findings were as follows: esophagogastroduodenoscopy (EGD) revealed possible gastritis, thyroid stimulating hormone was mildly elevated (9 U/mL), and anti-thyroid peroxidase antibody was elevated at 50 mg/L. She was evaluated by a psychiatrist who prescribed sertraline, as well as recommended relaxation techniques; however, her family requested further evaluation, and she was transferred to our institution. On physical exam, she had disorganized high amplitude conjugate horizontal movements of her eyes which persisted with eye closure, severe truncal ataxia that prevented her from sitting up without support, and distinct abdominal myoclonus. Otherwise, her neurological exam, including detailed mental status exam testing, was unremarkable.

Symptomatic and empiric therapy with lorazepam, levetiracetam, and a 5-day course of high dose IV methylprednisolone was immediately initiated. Further work-up revealed a lymphocytic pleocytosis in her cerebrospinal fluid (CSF) of 25 WBCs, with an otherwise unremarkable profile. Cytology was negative for malignancy and flow cytometry demonstrated a T cell dominant inflammatory process believed to be reactive. Paraneoplastic panel was negative in the CSF; however, voltage-gated potassium channel antibodies (VGKC) were detected in her serum (0.05 nmol/L; normal <0.02 nmol/L). Notably, the CSF VGKC assay was not yet available in our laboratory at the time of this patient's evaluation. Review of outside duodenal biopsy slides obtained during EGD was consistent with acute* H. Pylori *infection.

**Figure 1:**
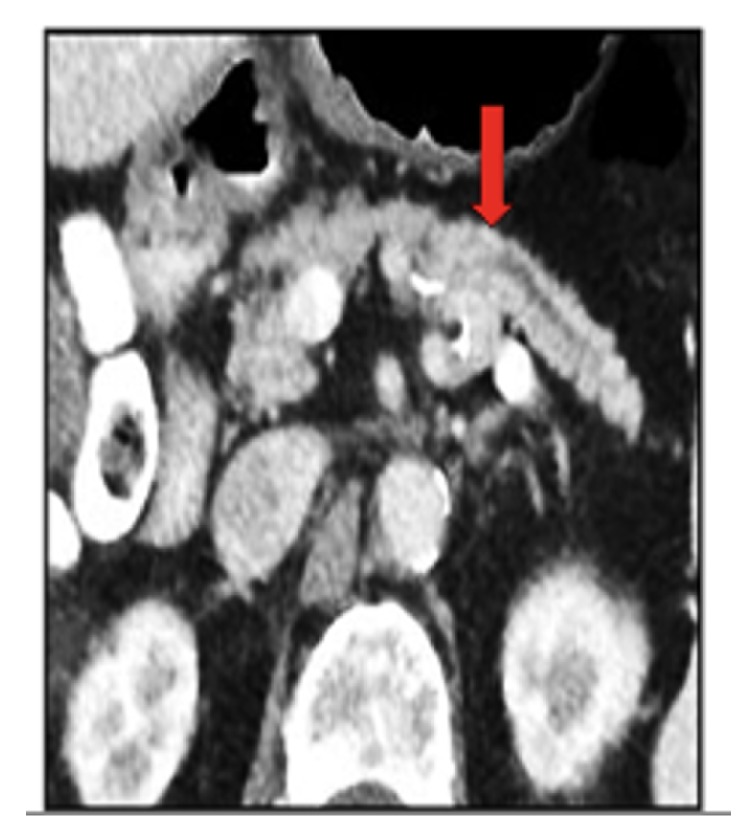
Axial abdominal CT-scan showing dilatation of the pancreas and duct cut-off sign (red arrow).

**Figure 2:**
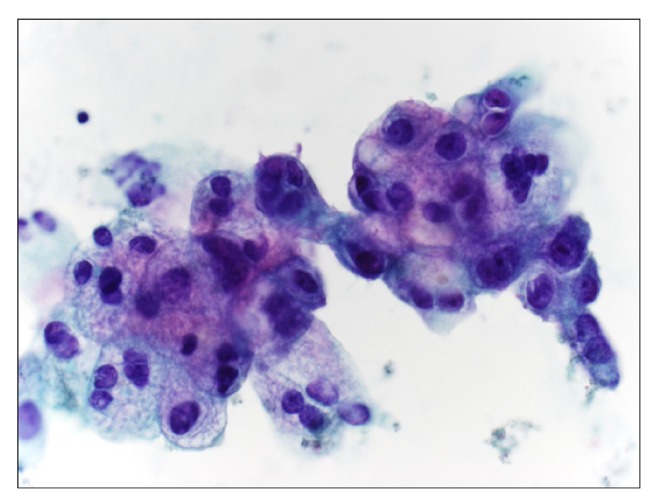
Endoscopic ultrasound (EUS) fine needle biopsy of pancreas revealed adenocarcinoma with mucinous features.

Computed tomography (CT) of the chest was normal. A subtle pancreatic duct cut-off sign was noted on abdominal CT, suggestive of a pancreatic mass (Figure 1). Subsequent MRI and endoscopic ultrasound-guided fine needle biopsy confirmed a ductal adenocarcinoma of the body of the pancreas (Figure 2). The patient underwent laparoscopic distal pancreatectomy and splenectomy, and a ductal adenocarcinoma, histologic grade 3/4 measuring 1.8 x 1.7 x 1.5 cm, in the body of the pancreas was removed. Negative operative margins were achieved, and all resected lymph nodes were negative for metastasis. Acutely following surgical resection, she experienced marked resolution of OMS symptoms. Upon taper and discontinuation of levetiracetam, some of her initial symptoms returned, including slight tremor and gait instability; however, the opsoclonus did not recur. Repeat serum paraneoplastic panel demonstrated VGKC normalization. She was initiated on single agent cisplatin-based chemotherapy and appeared to be responding well; however, she unfortunately died 7 months later, due to recurrent lung infections and pulmonary compromise.

## 3. Discussion

While the cellular origin of OMS is unclear, the cause of this dysfunction is likely autoimmune in nature. Although classic cases of OMS describe anti-Ri antibody (otherwise known as anti-neuronal nuclear autoantibody type 2 or ANNA2) as the biological culprit, the presence of several other anti-neuronal and surface antibodies has been confirmed, and these antibodies may be the mediators of motor neuron dysfunction that causes OMS [2, 4, 10–12]. VGKC, for example, the surface antibody in our patient, is a complex linked to the neuronal cell surface, and it is typically associated with limbic encephalitis or Morvan's syndrome, defined by neuropsychiatric symptoms and muscle hyperexcitability [13, 14]. To our knowledge, this is the first reported case of adult onset OMS associated with VGKC.

In both children and adults, OMS is often an indicator of an occult malignancy. In children, OMS is most commonly associated with neuroblastoma; however, in adults, OMS indicates the presence of cancer of the lung, breast, ovary, thyroid, or Hodgkin's Lymphoma [2, 15]. There is no current diagnostic test for OMS because the antigen responsible for initiating OMS has not been identified yet. Hence, the diagnosis of OMS is often clinical. The presence of involuntary, saccadic eye movements, ataxia, gait incoordination, rigidity, and tremor, and the detection of surface or onconeural antibodies in serum or CSF are extremely useful in diagnosing paraneoplastic OMS [15]. However, it is also common for OMS symptoms to be observed prior to cancer diagnosis, as was the case in our patient.

Treatment of OMS relies heavily on understanding the pathogenesis of the disease process. In the case of occult malignancy OMS, removal of the tumor, immunotherapy, and symptomatic management can be beneficial, especially if they are initiated in the early stages of symptom presentation, rather than once deficits are well established [16, 17]. The resolution of our patient's opsoclonus, improvement of her ataxia, and normalization of her neuronal antibodies following resection further support the hypothesis that her OMS was of paraneoplastic origin.

This patient is unique in that she presented with typical symptoms of OMS, yet her symptoms were caused by an underlying pancreatic adenocarcinoma, rather than a more commonly associated malignancy (e.g., lung cancer, breast cancer, and neuroblastoma). Importantly, her pre-clinical paraneoplastic symptoms provoked an aggressive malignancy screen, revealing pancreatic dilatation with a duct cut-off sign on CT. Upon resection, the patient's tumor was only 18 mm in greatest diameter. At this small size, the pancreatic mass is a subtle finding on CT and MRI and is only perceptible due to its obstruction of the main pancreatic duct, due to an abrupt duct cut-off sign [18, 19]. Presence of a duct cut-off sign should therefore prompt the clinician to pursue a more invasive malignancy search, as in our case with endoscopic biopsy.

Taken together, we highlight a rare case of OMS with an unusual presentation manifested as a result of pancreatic malignancy. Not only is this association with pancreatic cancer atypical, this case emphasizes and reviews the importance of the duct cut-off sign as an indicator for underlying pancreatic malignancy and highlights the next appropriate step to ensure accurate structural evaluation when the duct cut-off sign is observed in a patient with paraneoplastic neurological symptoms.
